# Developmental stage related patterns of codon usage and genomic GC content: searching for evolutionary fingerprints with models of stem cell differentiation

**DOI:** 10.1186/gb-2007-8-3-r35

**Published:** 2007-03-12

**Authors:** Lichen Ren, Ge Gao, Dongxin Zhao, Mingxiao Ding, Jingchu Luo, Hongkui Deng

**Affiliations:** 1 College of Life Sciences, Shanghai Jiao Tong University, Shanghai, 200240, PR China; 2 Center for Bioinformatics, College of Life Sciences, National Laboratory of Protein Engineering and Plant Genetics Engineering, Peking University, Beijing, 100871, PR China; 3 Department of Cell Biology and Genetics, College of Life Sciences, Peking University, Beijing, 100871, PR China

## Abstract

**Background:**

The usage of synonymous codons shows considerable variation among mammalian genes. How and why this usage is non-random are fundamental biological questions and remain controversial. It is also important to explore whether mammalian genes that are selectively expressed at different developmental stages bear different molecular features.

**Results:**

In two models of mouse stem cell differentiation, we established correlations between codon usage and the patterns of gene expression. We found that the optimal codons exhibited variation (AT- or GC-ending codons) in different cell types within the developmental hierarchy. We also found that genes that were enriched (developmental-pivotal genes) or specifically expressed (developmental-specific genes) at different developmental stages had different patterns of codon usage and local genomic GC (GCg) content. Moreover, at the same developmental stage, developmental-specific genes generally used more GC-ending codons and had higher GCg content compared with developmental-pivotal genes. Further analyses suggest that the model of translational selection might be consistent with the developmental stage-related patterns of codon usage, especially for the AT-ending optimal codons. In addition, our data show that after human-mouse divergence, the influence of selective constraints is still detectable.

**Conclusion:**

Our findings suggest that developmental stage-related patterns of gene expression are correlated with codon usage (GC3) and GCg content in stem cell hierarchies. Moreover, this paper provides evidence for the influence of natural selection at synonymous sites in the mouse genome and novel clues for linking the molecular features of genes to their patterns of expression during mammalian ontogenesis.

## Abstract 2

Developmental-stage-related patterns of gene expression correlate with codon usage and genomic GC content in stem cell hierarchies.

## Background

Synonymous codons, which encode the same amino acid, are not used randomly. Such codon usage biases are explained as the balance between mutational drift and natural selection [1]. In unicellular organisms [2-6] and invertebrate metazoans [7-11], the levels of gene expression can be used to interpret their codon biases. Specifically, highly expressed genes, compared with weakly expressed ones, selectively use 'optimal codons' that correspond to abundant tRNAs so as to improve their translational efficiency [11-15].

Nevertheless, in vertebrates, whose genes display more dramatic codon usage biases than those of simple organisms [14], the correlations between codon usage and patterns of gene expression (that is, the levels and breadth of gene expression) remain a subject of controversy [11,16]. In a number of rodent and human tissues, recent studies have indicated positive correlations between levels of gene expression, as estimated by SAGE and/or microarray analysis, and GC3 [16-19]. However, these results are in contradiction with observations made by analyzing expressed sequence tags (ESTs) [11,16]. Among extremely highly expressed genes, the H3 histone gene family is biased to use GC-ending codons [20]. However, there is no difference in codon usage between ribosomal protein genes, which are also expressed at very high levels, and other genes [14]. As to correlations between breadth of gene expression and codon usage, some studies suggest that housekeeping genes, with a wider breadth of expression, are biased to use GC-ending codons [18,21-24] (also see the debate between [25] and [16]); however, other papers have described different observations [11,26-29]. Although codon usage has been found to exhibit variations in human genes specifically expressed in six tissues [30], the effect is very weak [31] and cannot be generalized to interpret the global variation (the preference of AT-ending or GC-ending codons) of synonymous codons in the thousands of mammalian genes.

Moreover, in vertebrates, the reasons why there are correlations between codon usage and patterns of gene expression remain to be elucidated. By using multivariance analyses (MVA), highly expressed genes have been observed to have excessive usage of T-ending codons in *Xenopus *[32] and the Cyprinidae family [33]. However, both natural selection and 'transcriptional associated mutation bias' (TAMB) [34-36] would account for these observations. In the tissues with no evidence of TAMB, a set of GC-ending codons favored in highly expressed genes has been suggested to be optimal codons [19]. Moreover, GC-ending codons are more abundant in highly expressed genes [18] and constitutively spliced exons [37]. However, if GC-ending codons are optimal due to selective advantages, it is difficult to see why the synonymous substitution rate (Ks) would be increased with GC-ending codon usage [38-41] or why the Ks of alternatively spliced exons would be lower than that of constitutively spliced exons [42]. It has been reported that highly expressed genes have higher recombination rates [43-45]. Moreover, according to the model of biased gene conversion (BGC), recombination rates are positively correlated with GC3 [46-51], indicating that both natural selection and BGC may be responsible for the correlations between the levels of gene expression and GC3. The variations of synonymous codon usage among tissue-specific genes have been suggested to be the consequence of translational selection [30]; a recent study, however, has indicated that these observations were due to regional variations of substitutional patterns rather than translational selection [31]. Taken together, further research is obviously still needed to explore the mechanisms of vertebrate codon usage bias.

**Figure 1:**
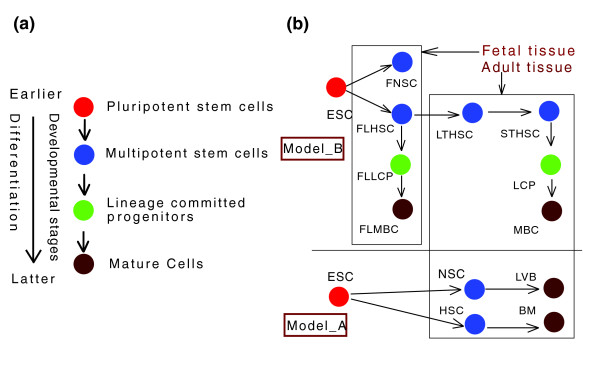
Cell types of different developmental stages in two models of stem cell differentiation. **(a) **Cell types of earlier developmental stages can differentiate into cell types of later developmental stages. The arrowheads indicate the direction of differentiation. Pluripotent stem cells (PSCs) occupy the earliest developmental stage, as they can give rise to all cell types of the three germ layers. PSCs can generate less potent 'multipotent stem cells' (MSCs), which are capable of generating all the cell lineages in specific tissues. MSCs can, in turn, give rise to lineage-committed progenitors (LCPs), which directly produce mature cells in the later developmental stage. **(b) **Two models of stem cell differentiation in our research. The cell type colors correspond to the developmental stages shown in (a). The arrows indicate the direction of differentiation within differentiation pairs made up of two neighboring stages in the developmental hierarchy. Model A [54] contains pluripotent embryonic stem cells (ESCs), MSCs in adult hematopoietic (hematopoietic stem cells (HSCs)) and neural (neural stem cells (NSCs)) tissues, as well as the main cell populations in bone marrow (BM) and the cells in lateral ventricles of the brain (LVB), which mainly contain mature cells in adult hematopoietic and neural tissues, respectively. Model B [55] contains ESCs and three types of MSCs that reside in fetal neural (fetal neural stem cell (FNSCs)), fetal liver hematopoietic (fetal liver hematopoietic stem cells (FLHSCs)) and adult hematopoietic (long-term functional hematopoietic stem cells (LTHSCs)) tissues. Model B also includes the key intermediate developmental stages of the hematopoietic hierarchy. In adult bone marrow, short-term functional HSCs (STHSC) and bone marrow LCPs are intermediate developmental stages in the course from LTHSCs to mature blood cells (MBCs). Fetal liver LCPs (FLLCPs) comprise an intermediate developmental stage between FLHSCs and FLMBCs. (For a detailed description of each cell type and experimental evidence of these differentiation processes, see Table 1 and Discussion).

In this paper, to investigate the regularity and mechanisms of mammalian codon usage, we have taken developmental stage-related patterns of gene expression into account in models of stem cell differentiation (Figure 1 and Table 1). Stem cells, progenitor cells and their derivates, defined by their distinct differentiation potential (Figure 1a), play critical roles in the early stages of metazoan ontogenesis and thus provide ideal models of the mammalian developmental hierarchy. Moreover, developmental processes are believed to be of critical importance to the investigation of evolutionary mechanisms [52], even at the genomic level [53]. In the current study, therefore, we have investigated the correlations between developmental stage-related patterns of gene expression and codon usage in developmental hierarchies of stem cell differentiation. Specifically, we have taken advantage of two independent models of stem cell differentiation [54,55] to identify developmental stage-related patterns of gene expression, as well as the correlations between these patterns of gene expression and codon usage.

**Table 1:** Descriptions and definitions of each cell type in the models of stem cell differentiation

Abbreviation	Model	Descriptions	Definitions
ESC	A	Pluripotent stem cell	C57Bl/6 cell line
NSC	A	Adult neural stem cell	*Neurosphere
LVB	A	Adult mature neural cell	Lateral ventricles of the brain
HSC	A	Long-term hematopoietic stem cell	^†^Lin^- ^c-Kit^+ ^Sca-1^+ ^CD34^- ^Hoe^low^
BM	A	Non-hematopoietic stem cell	Bone marrow main population
ESC	B	Pluripotent stem cell	CCE cell line
FNSC	B	Fetal neural stem cell	*^†^Hoe^low ^from neurosphere
FLHSC	B	Fetal liver hematopoietic stem cell	^†^Lin^- ^AA4.1^+ ^c-Kit^+ ^Sca-1^+^
FLLCP	B	Fetal liver hematopoietic progenitor cell	^†^Lin^- ^AA4.1^+ ^c-Kit^+ ^Sca-1^-^
FLMBC	B	Fetal liver mature blood cell	^†^Lin^+^
LTHSC	B	Long-term hematopoietic stem cell	^†^Lin^- ^c-Kit^+ ^Sca-1^+^Rho^low^
STHSC	B	Short-term hematopoietic stem cell	^†^Lin^-^c-Kit^+ ^Sca-1^+^Rho^high^
LCP	B	Hematopoietic progenitor cell	^†^Lin^-^c-Kit^+ ^Sca-1^-^
MBC	B	Mature blood cell	^†^Lin^+^
CD45	B	Contain long-term hematopoietic stem cells	^†^CD45^+ ^c-Kit^+ ^Sca-1^+^

Stem cells and progenitor cells are defined in terms of their surface markers (^†^by FACS sorting) and/or growth characters (*by selective culture). ESCs in both models A and B were functionally tested. For detailed descriptions and related references, see [54,55].

To define the developmental stage-related patterns of gene expression in models of stem cell differentiation, we have introduced two parameters. First, the 'level of gene expression' has been defined as the intensity of gene transcription in a particular cell type. Second, the 'fold change of gene expression' has been defined as the ratio of the expression levels of the same gene in two cell types of two neighboring stages in the developmental hierarchy (Figure 1b). We have further defined one of these two cell types, in the upper developmental hierarchy, as the earlier cell type, and the other, in the lower developmental hierarchy, as the later cell type. These two cell types together constitute a 'differentiation pair'. Thus, the 'fold change of gene expression' is a descriptive index of the levels of gene enrichment in a given differentiation pair.

In the present work, we investigate the correlations between developmental stage-related patterns of gene expression (that is, the 'levels of gene expression' in each cell type in the models of stem cell differentiation and the 'fold changes of gene expression' in each differentiation pair) and the molecular features (GC3 and genomic GC (GCg) content) of these genes. We also explore possible mechanisms for these developmental stage-related patterns of codon usage. This study reveals that developmental stage-related patterns of gene expression are correlated with GC3 and GCg in models of stem cell differentiation. Moreover, these analyses suggest that the model of translational selection, rather than other known hypotheses that have been put forward, might be the most likely to account for the developmental stage-related patterns of codon usage, especially for the negative correlations between the levels of gene expression and GC3.

## Results

### 'Levels of gene expression' are correlated with GC3 and GCg: variation of optimal codons within developmental hierarchies

First, we focused on the correlations between the levels of gene expression and GC3. We found significant negative correlations between the levels of gene expression and GC3 in eight cell types (*P *< 0.005; Table 2). In these datasets, we observed that only in the lateral ventricles of the brain (LVB), which contain predominantly mature neural cells, were the levels of gene expression significantly positively correlated with GC3 (*P *< 0.005; Table 2). We next investigated the variation of codon usage between 'highly expressed genes' and 'mid to lowly expressed genes', which were divided by quantiles of 0.67 (Q_0.67_) of the levels of gene expression in each cell type. We observed that in the eight cell types in which the levels of gene expression were negatively correlated with GC3, the highly expressed genes used significantly more AT-ending codons compared with the mid to lowly expressed genes (*P *< 0.01; Table 2). In addition, in LVB, highly expressed genes used more GC-ending codons than mid to lowly expressed genes (*P *< 0.05; Table 2). The 'optimal codons' are defined here as the codons that were preferentially present in highly expressed genes. Our observations, therefore, show that the optimal codons vary within the developmental hierarchies.

**Table 2:** The levels of gene expression are correlated with GC3 and GCg

Cell (model)	Rs*	EXP	GC3^†^	GCg^†^	Ka^#^	Ks^#^	Ka/Ks^#^	Ks_noDS^#^
ESC (A)	-0.166^‡^	H	0.537^‡^	0.442^‡^	0.042^‡^	0.542^‡^	0.069^‡^	0.558^‡^
		M_L	0.580	0.452	0.057	0.573	0.090	0.604
NSC (A)	-0.098^‡^	H	0.551^‡^	0.446^‡^	0.039^‡^	0.537^‡^	0.067^‡^	0.555^‡^
		M_L	0.581	0.453	0.054	0.572	0.089	0.604
HSC (A)	0.010 (0.65)	H	0.579 (0.26)	0.456 (0.20)	0.052^§^	0.558 (0.10)	0.084^§^	0.581^¶^
		M_L	0.582	0.455	0.057	0.572	0.090	0.602
LVB (A)	0.056^§^	H	0.583^¶^	0.455^§^	0.042^‡^	0.541^‡^	0.070^‡^	0.568^‡^
		M_L	0.575	0.451	0.054	0.570	0.088	0.601
BM (A)	0.036 (0.09)	H	0.578 (0.38)	0.456^¶^	0.054^‡^	0.564 (0.19)	0.087^‡^	0.577^§^
		M_L	0.577	0.453	0.057	0.572	0.093	0.605
ESC (B)	-0.112^‡^	H	0.550^‡^	0.447^‡^	0.042^‡^	0.555^¶^	0.069^‡^	0.573^‡^
		M_L	0.583	0.453	0.061	0.568	0.098	0.604
FNSC (B)	0.014 (0.45)	H	0.573 (0.31)	0.452 (0.27)	0.040^‡^	0.546^§^	0.066^‡^	0.565^‡^
		M_L	0.574	0.451	0.056	0.568	0.091	0.601
FLHSC (B)	-0.108^‡^	H	0.551^‡^	0.447^§^	0.047^‡^	0.554^§^	0.077^‡^	0.567^‡^
		M_L	0.581	0.452	0.062	0.574	0.098	0.607
FLLCP (B)	-0.120^‡^	H	0.557^‡^	0.450^§^	0.047^‡^	0.558^¶^	0.075^‡^	0.572^‡^
		M_L	0.585	0.453	0.062	0.573	0.100	0.608
FLMBC (B)	-0.109^‡^	H	0.548^‡^	0.447^§^	0.047^‡^	0.547^‡^	0.078^‡^	0.563^‡^
		M_L	0.579	0.451	0.062	0.580	0.098	0.613
LTHSC (B)	0.041 (0.07)	H	0.559 (0.23)	0.446 (0.12)	0.049^‡^	0.547^§^	0.081^§^	0.560^§^
		M_L	0.555	0.443	0.055	0.570	0.089	0.593
STHSC (B)	0.015 (0.50)	H	0.552 (0.15)	0.445 (0.48)	0.048^‡^	0.550^§^	0.078^‡^	0.558^‡^
		M_L	0.558	0.445	0.056	0.571	0.091	0.597
LCP (B)	-0.092^§^	H	0.546^‡^	0.444^‡^	0.048^‡^	0.557^¶^	0.077^‡^	0.569^‡^
		M_L	0.573	0.450	0.058	0.570	0.096	0.602
MBC (B)	-0.056^§^	H	0.558^§^	0.446^§^	0.053^‡^	0.555^§^	0.084^‡^	0.570^‡^
		M_L	0.572	0.450	0.059	0.573	0.096	0.606
CD45 (B)	-0.003 (0.87)	H	0.579 (0.35)	0.455^¶^	0.050^‡^	0.560 (0.09)	0.080^‡^	0.577^‡^
		M_L	0.580	0.452	0.063	0.572	0.101	0.610

*Rs: Spearman correlation coefficients between EXP (the levels of gene expression) and GC3 (^‡^*P *< 5 × 10^-6^, ^§^*P *< 0.005). *P *values are shown if there was no significance (*P *> 0.05). ^†^Wilcoxon test was used to determine whether GC3 and GCg of highly expressed genes (H) were lower (or higher) than GC3 and GCg of mid to lowly expressed genes (M_L). Highly expressed genes and mid to lowly expressed genes were divided by quantiles of 0.67 (Q_0.67_) of the levels of gene expression (^‡^*P *< 0.001, ^§^*P *< 0.01, ^¶^*P *< 0.05). *P *values are shown if there was no significance (*P *> 0.05). ^#^Wilcoxon test was used to determine whether Ka, Ks, Ka/Ks and Ks_noDS of highly expressed genes (H) were lower than Ka, Ks, Ka/Ks and Ks_noDSof mid to lowly expressed genes (M_L) (^‡^*P *< 0.001, ^§^*P *< 0.01, ^¶^*P *< 0.05). *P *values are shown if there was no significance (*P *> 0.05).

In accordance with the variation in GC3, we found that GCg was also significantly different between highly expressed genes and mid to lowly expressed genes in each of the nine cell types (*P *< 0.05), where the levels of gene expression were significantly correlated (positively in LVB or negatively in the eight cell types) with GC3 (*P *< 0.005; Table 2). Consistent with earlier studies (for example, [14,40]), we observed that GC3 and GCg were closely correlated in our dataset (Spearman rank correlation coefficient (*Rs*) = 0.665, *N *= 11,066; *P *< 10^-6^). We thus suggest that the variation of GCg between the highly expressed and mid to lowly expressed genes might well be a consequence of this correlation.

### 'Fold changes of gene expression' are correlated with GC3 and GCg: genes specifically expressed in different developmental stages bear different molecular features

First, we established correlations between the fold changes of gene expression and GC3 in 12 differentiation pairs for which there was experimental evidence of the differentiation processes (Figure 1b; also see Discussion). We found that in 10 of the 12 differentiation pairs, the fold changes of gene expression were significantly correlated with GC3 (*P *< 0.005; Table 3). Strikingly, in differentiation pairs of neural stem cells (NSCs)/LVB and embryonic stem cells (ESCs)/hematopoietic stem cells (HSCs), up to 14.3% (*Rs *= 0.378) and 11.4% (*Rs *= 0.338) variation of GC3 could be explained by the respective fold changes of gene expression in these differentiation pairs.

**Table 3:** Fold changes of gene expression are correlated with GC3 and GCg

DP* (model)	*Rs*^†^	Class		GC3^‡^	GCg^‡^	Ka^§^	Ks^§^	Ka/Ks^§^	Ks_noDS^§^
ESC/NSC (A)	-0.175^¶^	DPG	FC > 2	0.522^¶^	0.437^¶^	0.049 (0.10)	0.555 (0.45)	0.084 (0.14)	0.584 (0.23)
			FC < 0.5	0.584	0.454	0.044 (0.30)	0.545 (0.13)	0.075 (0.27)	0.580 (0.33)
		DSG	ESC	0.607^¥^	0.448^¶^	0.097^¶^	0.627^¶^	0.136^¶^	0.703^¶^
			NSC	0.642	0.469	0.058^#^	0.563 (0.29)	0.101^#^	0.619^¥^
		NDPG		0.557	0.448	0.048	0.561	0.079	0.577
NSC/LVB (A)	-0.378^¶^	DPG	FC > 2	0.510^¶^	0.433^¶^	0.042^#^	0.548^¥^	0.074^¥^	0.570 (0.15)
			FC < 0.5	0.636	0.461	0.042 (0.27)	0.549 (0.25)	0.069 (0.24)	0.589 (0.49)
		DSG	NSC	0.580^¶^	0.453 (0.10)	0.050 (0.28)	0.583 (0.16)	0.068 (0.35)	0.632^¥^
			LVB	0.635	0.462	0.081^¶^	0.592^#^	0.123^¶^	0.652^¶^
		NDPG		0.587	0.457	0.049	0.562	0.081	0.585
ESC/HSC (A)	-0.338^¶^	DPG	FC > 2	0.505^¶^	0.431^¶^	0.042^#^	0.542^#^	0.072^¥^	0.565^¥^
			FC < 0.5	0.610	0.469	0.052 (0.17)	0.547 (0.12)	0.082 (0.13)	0.583 (0.45)
		DSG	ESC	0.596^¶^	0.447^¶^	0.074^¶^	0.603^#^	0.107^¶^	0.663^¶^
			HSC	0.646	0.472	0.086^¶^	0.593^¥^	0.133^¶^	0.647^¶^
		NDPG		0.579	0.456	0.050	0.566	0.080	0.587
HSC/BM (A)	0.043 (0.08)	DPG	FC > 2	0.592^¥^	0.459 (0.08)	0.065^¶^	0.590^#^	0.099^¶^	0.627^#^
			FC < 0.5	0.565	0.451	0.067 (0.05)	0.576 (0.20)	0.107 (0.05)	0.609 (0.13)
		DSG	HSC	0.638^#^	0.473^#^	0.070^¶^	0.567 (0.25)	0.104^#^	0.639^#^
			BM	0.593	0.454	0.096^¶^	0.615^#^	0.148^¶^	0.670^#^
		NDPG		0.575	0.454	0.049	0.562	0.080	0.584
ESC/FNSC (B)	-0.238^¶^	DPG	FC > 2	0.528^¶^	0.442^¶^	0.045 (0.50)	0.560 (0.42)	0.081 (0.42)	0.590 (0.31)
			FC < 0.5	0.598	0.454	0.053^¥^	0.550 (0.19)	0.088^¥^	0.580 (0.36)
		DSG	ESC	0.599^¶^	0.455 (0.13)	0.083^¶^	0.591^¶^	0.125^¶^	0.642^¶^
			FNSC	0.635	0.460	0.062^¶^	0.573 (0.06)	0.100^¶^	0.630^¶^
		NDPG		0.569	0.452	0.049	0.561	0.079	0.585
ESC/FLHSC (B)	-0.003 (0.90)	DPG	FC > 2	0.566 (0.50)	0.450 (0.09)	0.046 (0.46)	0.576^¥^	0.073 (0.36)	0.606^#^
			FC < 0.5	0.571	0.447	0.060^#^	0.570 (0.08)	0.099^#^	0.600^¥^
		DSG	ESC	0.608 (0.32)	0.456 (0.44)	0.072^¶^	0.571^¥^	0.113^¶^	0.625^¶^
			FLHSC	0.617	0.455	0.097^¶^	0.599^¶^	0.143^¶^	0.640^¶^
		NDPG		0.562	0.450	0.051	0.557	0.082	0.579
FLHSC/FLLCP (B)	-0.058^#^	DPG	FC > 2	0.572 (0.17)	0.446^¥^	0.068 (0.08)	0.563 (0.44)	0.107 (0.06)	0.607 (0.16)
			FC < 0.5	0.587	0.458	0.069^¥^	0.594^¥^	0.104 (0.07)	0.629^¥^
		DSG	FLHSC	0.600^¥^	0.447^#^	0.082^¶^	0.564 (0.49)	0.123^¶^	0.590 (0.50)
			FLLCP	0.624	0.460	0.068^¶^	0.570 (0.28)	0.110^¶^	0.614^¥^
		NDPG		0.568	0.450	0.054	0.566	0.087	0.590
FLLCP/FLMBC (B)	0.108^¶^	DPG	FC > 2	0.602^#^	0.459^#^	0.062^#^	0.602^¶^	0.096^¥^	0.631^¶^
			FC < 0.5	0.575	0.449	0.068^¶^	0.566 (0.27)	0.107^¶^	0.616^¥^
		DSG	FLLCP	0.631^¶^	0.465^¶^	0.075^¶^	0.581^#^	0.116^¶^	0.634^¶^
			FLMBC	0.594	0.448	0.088^¶^	0.600^¶^	0.135^¶^	0.652^¶^
		NDPG		0.559	0.448	0.051	0.559	0.083	0.580
FLHSC/LTHSC (B)	-0.136^¶^	DPG	FC > 2	0.537^¶^	0.445 (0.82)	0.055 (0.14)	0.578 (0.06)	0.084 (0.41)	0.583 (0.29)
			FC < 0.5	0.587	0.445	0.075^¶^	0.567 (0.14)	0.108^¶^	0.603^¥^
		DSG	FLHSC	0.606 (0.77)	0.463^¶^	0.066^¶^	0.579^#^	0.103^¶^	0.622^¶^
			LTHSC	0.607	0.439	0.074^¶^	0.586 (0.05)	0.112^¶^	0.620^¥^
		NDPG		0.552	0.444	0.048	0.557	0.081	0.577
LTHSC/STHSC (B)	0.086^#^	DPG	FC > 2	0.567 (0.24)	0.437 (0.22)	0.084^¥^	0.563 (0.26)	0.128 (0.05)	0.632^¥^
			FC < 0.5	0.536	0.448	0.071^¶^	0.614^#^	0.107^#^	0.634^¥^
		DSG	LTHSC	0.590 (0.92)	0.446^#^	0.063^#^	0.564 (0.31)	0.106^¶^	0.595 (0.16)
			STHSC	0.585	0.459	0.066^¶^	0.575 (0.13)	0.109^¶^	0.618^¥^
		NDPG		0.553	0.444	0.051	0.560	0.082	0.577
STHSC/LCP (B)	0.141^¶^	DPG	FC > 2	0.599^¶^	0.449 (0.16)	0.076^¶^	0.562 (0.43)	0.123^¶^	0.615 (0.05)
			FC < 0.5	0.544	0.441	0.070^#^	0.590 (0.06)	0.106^#^	0.606 (0.14)
		DSG	STHSC	0.599 (0.99)	0.455 (0.44)	0.083^¶^	0.610^#^	0.104^#^	0.605 (0.13)
			LCP	0.600	0.459	0.063^¶^	0.578^¥^	0.103^¶^	0.617^¶^
		NDPG		0.552	0.445	0.050	0.560	0.082	0.580
LCP/MBC (B)	-0.081^#^	DPG	FC > 2	0.542^¶^	0.443^¥^	0.054^¥^	0.580^¥^	0.086 (0.12)	0.596 (0.16)
			FC < 0.5	0.584	0.450	0.066^#^	0.570 (0.19)	0.103^#^	0.610^¥^
		DSG	LCP	0.606 (0.68)	0.456 (0.64)	0.072^¶^	0.589^#^	0.120^¶^	0.626^#^
			MBC	0.610	0.457	0.081^¶^	0.590^#^	0.124^¶^	0.634^¶^
		NDPG		0.560	0.448	0.051	0.558	0.084	0.582

*DP, differentiation pairs. ^†^*Rs: *Spearman correlation coefficients (*Rs*) between FC (the fold change of gene expression) and GC3 (^¶^*P *< 5 × 10^-6^, ^#^*P *< 0.005). *P *values are still shown if there was no significance (*P *> 0.05). ^‡^Wilcoxon test was used to determine whether GC3 and GCg were different in a particular differentiation pair between developmental-pivotal genes (DPGs) enriched in earlier (FC > 2) and later (FC < 0.5) stages, as well as between developmental-specific genes (DSGs) expressed in the earlier and later stages (for example, FNSC refers to developmental specific genes in FNSC of differentiation pair ESC/FNSC (^¶^*P *< 0.001, ^#^*P *< 0.01, ^¥^*P *< 0.05). *P *values are shown if there was no significance (*P *> 0.05). ^§^Wilcoxon test was used to determine whether Ka, Ks, Ka/Ks and Ks_noDS of DPGs and DSGs were higher (or lower) than Ka, Ks, Ka/Ks and Ks_noDS of non-developmental-pivotal genes (NDPGs) (^¶ ^*P *< 0.001, ^#^*P *< 0.01, ^¥^*P *< 0.05). *P *values are still shown if there was no significance (*P *> 0.05).

We next investigated the variation of GC3 and GCg between genes enriched in two cell types of each differentiation pair. When genes are expressed in both cell types of a given differentiation pair, the 'fold change of gene expression' is a measurement of the level of gene enrichment in this differentiation pair. Thus, if the fold change of a certain gene expression is higher than 2 or less than 0.5, this gene is defined as a developmental-pivotal gene in this paper. Our results show that, in nine differentiation pairs, GC3 between the developmental-pivotal genes enriched at the earlier and later developmental stages differed significantly (*P *< 0.05; Table 3). Moreover, we also found GCg between these two groups of genes to be significantly different in seven differentiation pairs (*P *< 0.05), especially in ESC/NSC, NSC/LVB, ESC/HSC, and ESC/fetal neural stem cells (FNSCs) (*P *< 0.001; Table 3).

It should be noted that some genes, which were only expressed in either the earlier or later developmental stages, cannot be described in terms of 'fold change of gene expression'. We have defined these genes as developmental-specific genes. We found that both GC3 and GCg were different between developmental-specific genes in seven differentiation pairs (*P *< 0.05; Table 3). In addition, at the same developmental stage, most groups of developmental-specific genes generally use more GC-ending codons and are located in genomic domains with higher GC content compared with developmental-pivotal genes (Table 3; Additional data file 1).

### Possible mechanisms of developmental stage-related codon usage: testing the hypotheses of BGC, TAMB and natural selection

We then attempted to investigate the mechanisms resulting in the patterns of developmental stage-related codon usage observed. In mammals, BGC, mutational bias, and natural selection have been suggested to account for the biased usage of synonymous codons [11,40].

The BGC model suggests a positive correlation between GC content (including GC3) and recombination rates [46-50]. We observed that GC3 was positively correlated with recombination rates in our datasets (*Rs *= 0.14, *N *= 10383, *P *< 10^-6^). In this paper, we established the correlations between GC3 and the patterns of gene expression. Therefore, to determine if the developmental stage-related patterns of codon usage are byproducts of the BGC effect, we further studied the correlations between the patterns of gene expression and recombination rates. No significant correlations between recombination rates and the levels of gene expression were observed (*Rs *range from -0.033 to 0.020, *P *> 0.10; Additional data file 2). The only exception was in fetal liver mature blood cells (FLMBCs; *Rs *= -0.043, *P *= 0.02), but this correlation coefficient was weaker than that between the levels of gene expression and GC3 in FLMBCs. In our datasets, the fold changes of gene expression were significantly correlated with recombination rates only in the differentiation pairs NSC/LVB and FLLCP/FLMBC (*Rs *= -0.083 and 0.062, respectively, *P *< 0.01; Additional data file 3). Moreover, these correlation coefficients were weaker than those between the fold changes of gene expression and GC3 in these differentiation pairs (Table 3). In other differentiation pairs, no significant correlations between the fold changes of gene expression and recombination rates were observed (*Rs *range from -0.045 to 0.034, *P *> 0.05; Additional data file 3). We also observed that the recombination rates of developmental-specific genes, with their excessive usage of GC-ending codons, were not significantly higher than those of non-development pivotal genes (the fold changes of gene expression are within 0.5 and 2) (data not shown). Taken together, our results suggest that the developmental stage-related patterns of codon usage are not byproducts of the BGC effect.

The model of mutational bias proposes that the codon bias is simply due to unbalanced base substitutions [15,56-60]. Transcriptional processes can increase the mutation frequency from cytidine (C) to thymine (T) and adenosine (A) to guanosine (G), because the single-stranded DNA that more frequently appears during the course of transcription is more sensitive to deamination [34-36]. This TAMB model thus predicts a positive correlation between the levels of gene expression and the T or G content. If TAMB is the only cause of the excessive usage of T-ending and G-ending codons in highly expressed genes, we would expect an increase in the T3/G3 (T/G content at the third codon position) and Ti/Gi (T/G content in the untranslated region) in parallel with the levels of gene expression. To evaluate the influence of TAMB, we measured the slopes of Ni (the nucleotide content in the untranslated regions) and N3 (the nucleotide content at the third codon position) with the levels of gene expression as the descriptive index of their increase rates. Our results show that although there was a parallel increase in G3 and Gi in LVB, the increase in T3 (with the slopes ranging from 5.38 to 10.60) was more rapid than the increase in Ti (with the slopes ranging from 1.86 to 5.03) in other cell types where the levels of gene expression were negatively correlated with GC3 (Additional data file 2). Moreover, the increase in C3 (in LVB) relative to the levels of gene expression was not due to the contribution of TAMB. Consequently, although these results cannot completely rule out a potential effect of TAMB, there is a strong suggestion that some factors other than TAMB are the primary cause underlying our observations.

Natural selection could act on mammalian genes, for example, highly expressed genes are reported to prefer shorter [19,61] and less introns [62], as well as cheaper amino acids [62] (however, see [19]). Natural selection could also influence mammalian codon usage biases [62-68], for example, at the levels of transcription [69,70], RNA processing [71-73], translation [19,62,74,75] and mRNA secondary structure [76], as well as at the protein level [77,78]. If codons are selected to improve transcriptional efficiency, there would be more GC-ending codons in highly expressed genes, as the conformation of DNA with a higher GC content would facilitate transcription [69,70]. Therefore, it is not likely that the excessive usage of AT-ending codons in highly expressed genes is a result of this effect. If certain codons have selective advantages of translational efficiency over other codons, these codons would be used more frequently in highly expressed than in weakly expressed genes. Therefore, the correlations between the levels of gene expression and codon usage seem to be consistent with this hypothesis. Taken together, it is more likely that the model of translational selection, rather than BGC or TAMB, would account for these findings, especially for the negative correlations between the levels of gene expression and GC3.

If the codon bias of highly expressed genes has undergone selective pressures, it would be useful to determine whether selective pressures were still effective after the human-mouse divergence. Assuming mutational rates are near homogeneous in the mammalian genome, there would be lower synonymous substitution rates (Ks) between human-mouse orthologous genes if selective pressure was still effective. Except for HSCs, bone marrow (BM) of model A and CD45 of model B, our results show that highly expressed genes had lower Ks compared with mid to lowly expressed genes in all other cell types (*P *< 0.05; Table 2). Previous studies have indicated that the substitution rates at nonsynonymous sites may indirectly affect silent substitution rates [79]. We thus removed the codons in which doublet substitutions occurred to recalculate synonymous substitution rates (Ks_noDS) [80]. The data show that, in each of the 15 cell types in the different developmental stages, highly expressed genes had lower Ks_noDS compared to mid to lowly expressed genes (*P *< 0.05; Table 2). Moreover, we also demonstrate that the nonsynonymous substitution rates (Ka) and Ka/Ks of highly expressed genes are significantly lower than those of mid to lowly expressed genes (*P *< 0.01; Table 2).

We next focused on the substitution rates of developmental-pivotal genes and developmental-specific genes. We found that the developmental-pivotal genes in the earlier developmental stages of ESC/HSC and NSC/LVB had lower Ks and Ka/Ks than non-developmental-pivotal genes (*P *< 0.05; Table 3). Moreover, developmental-pivotal genes in the earlier developmental stages of ESC/HSC had lower Ks_noDS after removal of doublet substitutions (*P *< 0.05; Table 3). These results suggest the possibility that negative selection following human and mouse divergence may still be detectable in terms of the codon usage of some groups of developmental-pivotal genes. Nevertheless, we also show that many groups of developmental-pivotal genes, as well as almost all groups of developmental-specific genes, have higher Ks, Ka/Ks and Ks_noDS compared with non-developmental-pivotal genes (Table 3).

## Discussion

### The models of stem cell differentiation are precise descriptions of developmental hierarchies of mammalian ontogenesis

In this paper, to investigate developmental-stage related patterns of mammalian codon usage, we used two models of stem cell differentiation to define the developmental-stage related patterns of gene expression. Here we suggest that the patterns of gene expression defined in these models are faithful reflections of developmental regulation. First, development, as a process of ontogenesis, can be divided into many stages according to the steps of cellular differentiation. In our models, distinct cell types within the processes of differentiation were isolated with high homogeneity by strategies of selective culture and fluorescence activated cell sorting (FACS) (Table 1). To identify the patterns of gene expression in early developmental stages, these strategies of cell isolation seem more precise than those used previously, which postulated that complete embryos represent 'early developmental stages' [26,81], because embryos in fact are a mixture of differentiated mature cells with undifferentiated stem cells. Second, in our models, the processes of stem cell differentiation (Figure 1b) were constructed according to published experimental evidence. The pluripotency of ESCs can be examined by injecting them into blastocysts to produce normal embryos [82-84]. ESCs are able to differentiate into multipotent stem cells (MSCs), including the MSCs in neural [85] and hematopoietic [86] tissues. Moreover, both FNSCs [87] and adult NSCs [88] are able to generate mature neural cells *in vitro *and *in vivo*, including neurons, astrocytes and oligodendrocytes. Furthermore, both fetal liver hematopoietic stem cells (FLHSCs) [89] and bone marrow HSCs (or long-term hematopoietic stem cells (LTHSCs)) [90] can functionally repopulate entire hematopoietic systems in recipients. In these repopulation processes, hematopoietic stem cells give rise to mature blood cells by generating lineage-committed progenitors (LCPs). Notably, in cell lineage tracing assays, FLHSCs have been observed to acquire the ability to directly generate LTHSCs during ontogenesis [91].

### Developmental stage-related patterns of codon usage: methodological artifacts or byproducts of other correlations?

In this study, we observed that developmental stage-related patterns of gene expression (that is, the 'levels of gene expression' and the 'fold changes of gene expression') were correlated with GC3. Here we suggest that neither the methodological bias of the microarray nor the effect of the correlations between gene length and GC3 substantially influence these observations. Methodological issues are involved in the correlations between the levels of gene expression and codon usage. The SAGE and microarray analysis methods introduce a risk of overestimating the levels of gene expression with high GC content [11,92]. Therefore, our observation of excessive usage of AT-ending codons in highly expressed genes is not due to a methodological bias of microarray analysis. On the contrary, the actual correlation coefficients between the levels of gene expression and AT-ending codon usage might be even higher. Correlations between patterns of gene expression and gene length have been reported in mammals [19,62]; therefore, it is necessary for us to identify whether the correlations between the patterns of gene expression and GC3 are byproducts of these correlations. We suggest that gene lengths do not substantially influence these observations because, in our datasets, the levels of gene expression were negatively correlated with the lengths of both transcripts (ranging from -0.182 to -0.084, *P *< 10^-6^) and coding sequences (ranging from -0.172 to -0.084, *P *< 10^-6^) (Additional data file 2), whereas the levels of gene expression were negatively correlated with GC3 in most cases (Table 2). Moreover, gene lengths do not substantially affect the correlations between the fold changes of gene expression and GC3. In each of nine of ten differentiation pairs in which these correlations exist with significance (positively or negatively), the correlations between the fold changes of gene expression and gene lengths were weaker than, or were opposite to, the correlations between the fold changes of gene expression and GC3 (Table 3; Additional data file 3).

### Analyses of codon usage within developmental hierarchies: implications for understanding of evolutionary issues

Developmental processes are believed to be useful guides to the exploration of evolutionary mechanisms [93]. One famous example is the Haeckel's hypothesis that ontogeny may recapitulate, to some extent, phylogeny. Although it is clear that we can not simply regard the early stages of mammalian development as simple organisms [94], in this paper, using models of stem cell differentiation covering early stages of mammalian ontogeny, certain useful clues about evolutionary issues at the molecular level have been obtained. Some of these clues, for instance, the correlations between the levels of gene expression and codon usage, are shown to be helpful to understanding the codon usage biases that occur in simple organisms [2-11]. In addition, stem cells are observed as the units of natural selection [95,96] and the origin of many types of cancer [97,98]. These observations suggest that stem cells might play critical roles during evolutionary processes. Here we suggest that considering patterns of gene expression in early stages of developmental hierarchies (that is, stem cells and progenitor cells) might lead to a better understanding of mammalian codon usage biases.

#### AT-ending optimal codons in early developmental stages

In this paper, we found that optimal codons displayed variation (AT-ending or GC-ending codons) in different cell types within the developmental hierarchy. The 'optimal codons' are defined here as those codons that are excessively used in highly expressed genes. It has long been assumed that, in certain vertebrates, the optimal codons, if they exist, are consistent with the major codons, which are, on average, used more frequently when taking all the known transcripts of a species into account [16,18,19,62]. Notably, our results show that, in some special circumstances, for example, in certain mouse stem cells and progenitor cells in early developmental stages of mammalian ontogeny, the optimal codons were the AT-ending ones, while the mouse major codons are the GC-ending ones (average GC3 content of mouse transcripts is 0.555, based on Ensembl build 26). The difference between our observations and previous results may be explained by the fact that the previous studies, suggesting that GC-ending codons are the optimal codons, defined the levels of gene expression as average levels of gene expression in whole tissues, or whole organisms in embryonic or adult stages, which actually contain a mixture of all cell types in different developmental stages [16,18,19,62]. These strategies thus mainly reflect the patterns of gene expression in mature cells, and may not allow accurate characterization of gene expression patterns in the early developmental stages because stem cells and progenitor cells only constitute a negligible fraction of the tissues.

Previous reports have indicated correlations between GC-content and the patterns of gene expression in both human and mouse [11,16-18,25,27,99,100]. Specifically, mouse GC3 content is positively correlated with levels of gene expression in many tissues. The *R*^2 ^(*R*^2^: the correlation coefficient of determination that indicates how much of the variability in codon usage can be "explained by" variation in the levels of gene expression) of these correlations is as high as 2.6% (Spleen) and 2.3% [18]. In this work, we show that the *R*^2 ^of the negative correlations between mouse GC3 and the levels of gene expression could reach as high as 2.8% (ESCs of model A). This value is comparable with previous observations [18]. Notably, in the models of stem cell differentiation, defining the 'fold change of gene expression' as a novel pattern of gene expression, we observed that the *R*^2 ^of correlations between GC3 and the fold changes of gene expression in NSC/LVB (*R*^2 ^= 14.3%), ESC/HSC (*R*^2 ^= 11.4%) and ESC/FNSC (*R*^2 ^= 5.7%) were higher than the *R*^2 ^of correlations between GC3 and other known patterns of gene expression tested in the other mouse microarray dataset [16,18]. In this dataset, the levels of gene expression were defined as the average levels in each of 45 tissues [101]. We further tested whether taking early developmental stages into consideration could improve the predictability of codon usage by means of gene expression. Using MVA, we found that the levels of gene expression explained 16.0% (in 5 cell types of model A) and 15.5% (in 10 cell types of model B) of GC3 variation. These values are much higher than the 8.8% obtained from the average levels of gene expression in each of the 45 tissues [101]. This difference between our and previous results suggests that the AT-ending optimal codons in the early developmental stages seem to be critical to the understanding of the regularity of codon usage.

#### Possible explanations for the correlations between GC3 and the levels of gene expression

It has been suggested that the model of translational selection cannot be used to explain mammalian codon usage [14,102]. Conversely, recent studies have presented evidence that translational selection might influence the synonymous sites of coding regions [19,62,74,75]. These recent findings also agree with the observations that synonymous changes could dramatically influence translational efficiency in mammalian cells [103-106]. In the present study, we tested the hypotheses of BGC, TAMB and natural selection specifically at the levels of transcription and translation to analyze the possible mechanisms behind the developmental stage-related patterns of codon usage. From our results it is suggested that natural selection at the translational level, compared to the other hypotheses tested in this paper, most probably accounts for the finding that the levels of gene expression are correlated with GC3 in many cell types.

If the usage of synonymous codons correlates with translational efficiency, there might be a selective pressure to choose the synonymous codon that matches the most abundant tRNA. In unicellular organisms and invertebrate metazoans, the optimal codons are in general correspondence with the abundant tRNAs of high copy number [11-14,80,107]. Moreover, in the case of mammals, the abundances of tRNAs are also assumed to correlate with their copy number [19,74]. However, based on this assumption, it would be difficult to understand why optimal codons display variation (AT-ending or GC-ending codons) in the same species. Although the biological bases of the variations of optimal codons remain an issue for further investigation, we hypothesize that one of the aspects of these pressures may be related to variations in specific biochemical environments, for example, the developmental stage-related modification patterns of tRNA molecules. It has been reported that biochemical modification at the wobble positions of tRNA molecules helps regulate their codon recognition preference [108-111]. For example, uridine modified by thiolation or 5-carboxymethylation exhibits a preference for A over G at the third position of the codon [112]. Moreover, developmental stage-related patterns of tRNA modification have been observed [113,114]. Taken together, we suggest that the developmental stage-related variation of optimal codons might be correlated with developmental stage-related patterns of tRNA modification.

#### Possible explanations for the correlations between GC3 and the fold change of gene expression

In this paper, we defined the 'fold change of gene expression' as the ratio of the expression levels of the same gene in two cell types from neighboring stages in the developmental hierarchy. It is not surprising that the correlations between the 'fold change of gene expression' and GC3, in specific differentiation pairs, are related to the correlations between the 'levels of gene expression' and GC3 in these two cell types. Moreover, if the correlations between the 'levels of gene expression' and GC3 are the consequence of natural selection, we would regard the correlations between the 'fold change of gene expression' and GC3 as a reflection of the difference between selective pressures in the cell types occupying earlier and later developmental stages. In the differentiation pairs ESC/NSC, NSC/LVB, ESC/HSC, ESC/FNSC, FLHSC/LTHSC and LCP/mature blood cells (MBCs), selective pressure towards AT-ending codons is much stronger in cell types of an earlier rather than a later developmental stage; the genes enriched in the earlier cell types will show a greater usage of AT-ending codons than those in later cell types. In short-term hematopoietic stem cells (STHSCs)/LCP, similar results were obtained. Consistent with the explanation above, in ESC/FLHSC, the selective pressures towards AT-ending codons are very similar between the cell types of earlier and later developmental stages, the patterns of codon usage between the genes enriched in the earlier and later developmental stages are not significantly different (Table 3). However, we observed that, in FLHSC/FLLCP, FLLCP/FLMBC, and LTHSC/STHSC, in which selective pressures towards AT-ending codons are very similar for the cell types of earlier and later developmental stages, the fold changes of gene expression were significantly correlated with AT3. We suggest that these observations may be attributed to the fact that the codon usage of many genes enriched in certain differentiation pairs is affected by other factors that contribute to the codon usage bias of this differentiation pair. Taken together, our observations are consistent with the possibility that the greater the differences between the putative selective pressures of the cell types occupying earlier and later developmental stages, the greater the variation in codon usage (GC3) between genes enriched in the earlier and latter cell types (Table 3). In the differentiation pairs, we also show that the GC3 of the genes that were highly expressed in both earlier and later developmental stages were correlated with the sum of the correlation coefficients between the levels of gene expression and GC3 in these two stages (that is, the putative combination of selective pressures; *Rs *= 0.78).

#### Comparative genomic analysis of developmental stage-related genes

We also provide evidence of the presence of negative selection at synonymous sites following the human-mouse divergence. The observation that, in all mouse cell types, highly expressed genes have a lower Ks_noDS (Ks after removing doublet substitution) is consistent with previous results showing that synonymous substitution rates are lower in highly expressed genes compared with other genes in bacteria and *Drosophila *[9,115-117]. Considering the occurrence of negative selection at synonymous sites, it is suggested that Ka/Ks, which have long been used to evaluate protein evolutionary rates, carry a risk of overestimation [64]. Therefore, early studies in which exonic synonymous sites have been assumed neutral may require reevaluation (also see [19,64,65]). Notably, even with lower Ks, highly expressed genes and developmental-pivotal genes in ESCs of the ESC/HSC differentiation pair still showed lower evolutionary rates (Ka/Ks; Tables 2 and 3). These findings are consistent with previous results that protein evolutionary rates are negatively correlated with levels of gene expression from unicellular organisms to vertebrates [118-120].

In many groups of developmental-pivotal and developmental-specific genes, we also show that both Ks and Ka/Ks are higher than in non-developmental-pivotal genes. These results suggest that the codon usage of most developmental-pivotal and developmental-specific genes has been under less selective constraints. Furthermore, the higher Ka/Ks of these genes may imply that these genes have been subject to different functional constraints after the divergence of human and mouse. This explanation is consistent with the observation that orthologous genes can play different roles in human and mouse stem cells [121]. However, it should be noted that current knowledge of the mechanisms of stem cell differentiation is very limited. Therefore, further study of the function of orthologous developmental-pivotal and developmental-specific genes will deepen our understanding of the higher Ks and Ka/Ks in these genes.

#### Comparisons between developmental-pivotal genes and developmental-specific genes

The expression of developmental-pivotal genes (regulated up and down) and developmental-specific genes (regulated on and off) is regulated by different strategies. After the combination of these two groups of genes, both GC3 and GCg still differed significantly between the genes selectively expressed at the earlier and later developmental stages of many differentiation pairs (Additional data file 4). However, our data show that these two groups of genes are different in their molecular characteristics, genomic composition and the related evolution rates. Therefore, in this paper, developmental-pivotal genes and developmental-specific genes are discussed separately.

First, compared with developmental-pivotal genes, developmental-specific genes used more GC-ending codons and were located in genomic regions with higher GC content in most cases (Table 3; Additional data file 1). Second, the Ka, Ks, Ks_noDS, and Ka/Ks for many groups of developmental-specific genes were significantly higher than those of the developmental-pivotal genes (Table 3; Additional data file 5). According to these observations, we suggest these two groups of genes are different. Although more evidence is clearly still necessary, the results suggest the possibility that the regulation patterns of genes might be correlated with their codon usage, genomic GC content and evolutionary rates.

### Analyses of codon usage within developmental models: implications for understanding differentiation processes

The current study has applied analyses of codon usage to processes of stem cell differentiation to gain a better understanding of developmental processes (that is, the processes of stem cell differentiation) at the genomic level [122]. First, both developmental-pivotal genes and developmental-specific genes have been proposed, and many of them are experimentally demonstrated, to be responsible for maintaining cells at each developmental stage as well as regulating cell differentiation processes [54,55]. We have shown that codon usage, a 'silent' property of both developmental-pivotal genes and developmental-specific genes, are different between the earlier and later developmental stages in differentiation pairs. These findings suggest that the genes responsible for different developmental stages have different derivations and regulation patterns. Moreover, developmental-pivotal genes and developmental-specific genes exhibit different regulation patterns. During differentiation, the transcriptional intensities of developmental-pivotal genes need to be appropriately regulated up or down, whereas the transcription of developmental-specific genes should be silenced in one stage and activated in another. It has been suggested that chromatin structures and the genome location of developmental-pivotal and developmental-specific genes are quite different: developmental-pivotal genes might be located in euchromatin, whereas most developmental-specific genes might be located in facultative heterochromatin [123]. In this paper, we demonstrate that developmental-specific genes generally use more GC-ending codons than developmental-pivotal genes. We suggest that this different molecular property may correlate with different regulation patterns and chromatin structure, but the precise mechanisms at the moment remain unclear.

Second, it has been shown that the processes of stem cell differentiation are accompanied by remodeling of the entire chromatin structure [123-128]. However, little is known about the characteristics of chromatin segments involved in these remodeling processes. Previous studies have shown that the chromatin segments in which developmental stage-specific genes are located have been remodeled during differentiation [129-132]. Moreover, it has been reported that nucleosome formation potential is correlated with the GC content of DNA [69]. Our results suggest that the GC content of genomic regions where developmental-pivotal genes and developmental-specific genes are located is different between the earlier and later developmental stages in differentiation pairs. Altogether, our results suggest that, during differentiation, the genome segments that are involved in chromatin remodeling are correlated with their GC content. It has been suggested that mammalian genomes are made up of mosaic 'isochore' structures, which might relate to the variation in GC content on the scale of hundreds of kilobases to megabases [22,23,40,133,134]. Furthermore, the isochores are proposed to correlate with tissue specificity [18]. Previous work also shows that, during ESC differentiation, many differentiation-induced replication-timing and expression changes are restricted to AT-rich isochores [135]. Our findings of developmental stage-correlated codon usage and GCg content indicate that the isochores are related to different developmental stages during mammalian ontogenesis.

## Conclusion

In this investigation, using models of stem cell differentiation, developmental stage-related patterns of mouse codon usage have been observed. Notably, in early stages of mouse ontogeny, we found a bias for AT-ending optimal codons. Moreover, during mammalian ontogenesis, we also found that genes selectively expressed during different developmental stages have different codon usage (GC3) and local GCg content. We hypothesize that translational selection, compared to other hypotheses such as BGC and TAMB, most probably accounts for these codon usage biases, especially for the AT-ending optimal codons. The selective constraints were still detectable at synonymous sites of many groups of developmental stage-related genes. Moreover, at the same developmental stage, we also found that developmental-specific genes usually used more GC-ending codons, had higher GCg content and higher substitution rates compared with developmental-pivotal genes. Applying codon usage analysis in developmental hierarchies, this paper provides new clues for understanding differentiation processes. For example, the genome segments that are involved in chromatin remodeling may correlate with GC content. Further investigation will be needed to better understand the significance and implications of the findings presented here.

## Materials and methods

### Genomic data

Removing 2,672 pseudo genes according to their annotations, we extracted information on 31,022 transcripts from the Mouse division (build 26) of the Ensembl genome database for further analysis. To investigate the evolutionary conservation of mouse genes, we also extracted information from the Human division (build 26) of the Ensembl database.

### Microarray data

We used two independent oligonucleotide microarray datasets (Affymetrix MG-U74Av2) for the models of mouse stem cell differentiation [54,55]. For dataset A, the raw data are available from the website of Melton's lab [136]. We processed these raw data by Affymetrix MAS 5.0. For dataset B, the raw data were processed by Affymetrix MAS 4.0 [55]. We accessed these data from Science website [137]. For both datasets, we used the 'Detection Call' provided by the Affymetrix MAS system to identify whether a transcript is present (P) or absent (A); the marginal situation is marked as M.

The mapping relationships between Affymetrix probe-sets and their corresponding transcripts were extracted from the Ensembl database. The detailed mapping algorithms were implemented by the Ensembl team [138].

For dataset A, we used the average levels of two replicates as the levels of gene expression, if the probe-sets fulfilled the following criteria. First, in both replicates, the gene was expressed stably such that the standard error (SE) was less than a quarter of the measured expression value:

$$\frac{SE}{Exp}\leq 1/4$$

Second, the gene expression levels were stable between two replicates such that the absolute value of difference between the two replicates' expression values is smaller than half of their mean value

$$\frac{\left|\exp1-\exp2\right|}{mean(\exp1,\exp2)}\leq 0.5$$

According to the data provided, in dataset B, the average levels of two to four replicates were used as the levels of gene expression. Moreover, genes with expression levels below 200 were removed to confirm gene expression as suggested by Su *et al*. [101].

To calculate the codon usage, only probe-sets corresponding to unique transcripts on U74Av2 were considered.

### Nucleotide composition analysis

The untranslated regions (UTRs) and coding sequences (CDSs) of a given transcript were extracted from the Ensembl database according to the entry's annotation and validated by chromosome mapping. Sequences with ambiguous annotations were checked manually. To evaluate the influence of TAMB on gene composition, we calculated the nucleotide content in UTRs and the third position of synonymous codons in CDS for A, C, G and T [19,36]. We also calculated nucleotide composition (GC fraction) in contiguous 20 kb windows, as suggested by Lercher *et al*. [100], as genomic background of a given gene (Tables 2 and 3)

### Recombination rate estimates

Recombination rates across the mouse genome were estimated by dividing the genetic length (cM) by the sequence length (Mb) between genetic markers [49,139]. These data were derived from The Whitehead Mouse Genetic Map website [140].

### Codon usage analyses

CodonW software was used to calculate the GC content at the third codon positions (GC3) and the RSCU value of each synonymous codon according to Sharp *et al*. [4]. Only genes with CDS > 200 were considered.

### Comparative genomics

We detected an orthologous relationship based on the Ensembl build 26 EnsMart Database's annotation. The Ka, Ks and Ka/Ks were calculated using Nei and Gojobori methods [141] using PAML (yn00) [142,143] for each ortholog pair. According to the PAML manual [144], we excluded genes with Ks > 1 for further analyses. Synonymous substitution rates after removing doublet substitutions (Ks_noDS) were calculated as previous described [80] (Tables 2 and 3).

### Statistical analysis

Spearman's correlation test was used for analysis of paired samples and linear regression analysis was performed by standard routines using the statistical package R [145]. All necessary scripts and/or programs are available.

## Additional data files

The following additional data are available with the online version of this paper. Additional data file 1 provides comparisons of GC3 and GCg between developmental-pivotal genes and developmental-specific genes. Additional data file 2 includes supplementary information about the mechanisms of our observations showing that levels of gene expression are correlated with codon usage, recombination rate, gene length and nucleotide composition. Additional data file 3 includes supplementary information about the mechanisms of our observations showing that the fold changes of gene expression are correlated with codon usage, recombination rate and gene length. Additional data file 4 provides results on the GC3 and GCg of developmental-pivotal genes, developmental-specific genes and both together in each differentiation pair. Additional data file 5 provides comparisons of substitution rates between developmental-pivotal genes and developmental-specific genes.

## Supplementary Material

Additional data file 1 Comparisons of GC3 and GCg between developmental-pivotal genes and developmental-specific genes Click here for file

Additional data file 2 Levels of gene expression are correlated with codon usage, recombination rate, gene length and nucleotide composition Click here for file

Additional data file 3 Fold changes of gene expression are correlated with codon usage, recombination rate and gene length Click here for file

Additional data file 4 GC3 and GCg of developmental-pivotal genes, developmental-specific genes and both together in each differentiation pair Click here for file

Additional data file 5 Comparisons of substitution rates between developmental-pivotal genes and developmental-specific genes Click here for file
