# Supplementary material for: Developmental stage related patterns of codon usage and genomic GC content: searching for evolutionary fingerprints with models of stem cell differentiation
Source: Genome Biol. 2007 Mar 12;8(3):R35. doi: 10.1186/gb-2007-8-3-r35 (PMC1868930; doi:10.1186/gb-2007-8-3-r35)
Supplement: Additional data file 1 — Comparisons of GC3 and GCg between developmental-pivotal genes and developmental-specific genes [file gb-2007-8-3-r35-S1.doc]

**Additional data file 1: The Comparisons of GC3 and GCg between DPG and DSG**

| **DP^1^/Model** | **Class** | | **GC3^2^** | **GCg^2^** | **RR^2^** |
| --- | --- | --- | --- | --- | --- |
| **ESC/NSC**  **(A)** | **ESC** | **DPG** | 0.522  *** | 0.437  * | 0.585  (0.26) |
|  |  | **DSG** | 0.607 | 0.448 | 0.486 |
|  | **NSC** | **DPG** | 0.584  *** | 0.454  *** | 0.466  (0.41) |
|  |  | **DSG** | 0.642 | 0.469 | 0.488 |
| **NSC/LVB**  **(A)** | **NSC** | **DPG** | 0.510  *** | 0.433  *** | 0.508  (0.38) |
|  |  | **DSG** | 0.580 | 0.453 | 0.466 |
|  | **LVB** | **DPG** | 0.636  (0.47) | 0.461  (0.34) | 0.584  ** |
|  |  | **DSG** | 0.635 | 0.462 | 0.470 |
| **ESC/HSC**  **(A)** | **ESC** | **DPG** | 0.505  *** | 0.431  *** | 0.551  (0.41) |
|  |  | **DSG** | 0.596 | 0.447 | 0.505 |
|  | **HSC** | **DPG** | 0.610  *** | 0.469  (0.20) | 0.550  * |
|  |  | **DSG** | 0.646 | 0.472 | 0.503 |
| **HSC/BM**  **(A)** | **HSC** | **DPG** | 0.592  *** | 0.459  *** | 0.533  (0.31) |
|  |  | **DSG** | 0.638 | 0.473 | 0.541 |
|  | **BM** | **DPG** | 0.565  * | 0.451  (0.32) | 0.558  (0.45) |
|  |  | **DSG** | 0.593 | 0.454 | 0.519 |
| **ESC/FNSC**  **(B)** | **ESC** | **DPG** | 0.528  *** | 0.442  *** | 0.489  (0.38) |
|  |  | **DSG** | 0.599 | 0.455 | 0.503 |
|  | **FNSC** | **DPG** | 0.598  *** | 0.454  * | 0.518  (0.21) |
|  |  | **DSG** | 0.635 | 0.460 | 0.524 |
| **ESC/FLHSC**  **(B)** | **ESC** | **DPG** | 0.566  *** | 0.450  ** | 0.488  (0.18) |
|  |  | **DSG** | 0.608 | 0.456 | 0.484 |
|  | **FLHSC** | **DPG** | 0.571  *** | 0.447  ** | 0.549  (0.16) |
|  |  | **DSG** | 0.617 | 0.455 | 0.521 |

**Additional data file 1 continued:**

| **FLHSC/FLLCP**  **(B)** | **FLHSC** | **DPG** | 0.572  * | 0.446  (0.42) | 0.457  (0.22) |
| --- | --- | --- | --- | --- | --- |
|  |  | **DSG** | 0.600 | 0.447 | 0.538 |
|  | **FLLCP** | **DPG** | 0.587  *** | 0.458  (0.31) | 0.515  (0.20) |
|  |  | **DSG** | 0.624 | 0.460 | 0.525 |
| **FLLCP/FLMBC**  **(B)** | **FLLCP** | **DPG** | 0.602  *** | 0.459  ** | 0.537  (0.33) |
|  |  | **DSG** | 0.631 | 0.465 | 0.512 |
|  | **FLMBC** | **DPG** | 0.575  * | 0.449  (0.49) | 0.483  (0.13) |
|  |  | **DSG** | 0.594 | 0.448 | 0.533 |
| **FLHSC/LTHSC**  **(B)** | **FLHSC** | **DPG** | 0.537  *** | 0.445  *** | 0.510  (0.24) |
|  |  | **DSG** | 0.606 | 0.463 | 0.530 |
|  | **LTHSC** | **DPG** | 0.587  (0.17) | 0.445  (0.14) | 0.506  (0.29) |
|  |  | **DSG** | 0.607 | 0.439 | 0.675 |
| **LTHSC/STHSC**  **(B)** | **LTHSC** | **DPG** | 0.567  (0.10) | 0.437  (0.13) | 0.532  (0.50) |
|  |  | **DSG** | 0.590 | 0.446 | 0.520 |
|  | **STHSC** | **DPG** | 0.536  *** | 0.448  ** | 0.504  (0.12) |
|  |  | **DSG** | 0.585 | 0.459 | 0.462 |
| **STHSC/LCP**  **(B)** | **STHSC** | **DPG** | 0.599  (0.44) | 0.449  (0.20) | 0.518  (0.26) |
|  |  | **DSG** | 0.599 | 0.455 | 0.584 |
|  | **LCP** | **DPG** | 0.544  *** | 0.441  *** | 0.447  (0.14) |
|  |  | **DSG** | 0.600 | 0.459 | 0.523 |
| **LCP/MBC**  **(B)** | **LCP** | **DPG** | 0.542  *** | 0.443  *** | 0.514  (0.40) |
|  |  | **DSG** | 0.606 | 0.456 | 0.494 |
|  | **MBC** | **DPG** | 0.584  *** | 0.450  ** | 0.486  * |
|  |  | **DSG** | 0.610 | 0.457 | 0.522 |

1 DP: differentiation pairs

2 Wilcoxon test was used to determine whether GC3, GCg and recombination rates (RR) of developmental-specific genes (DSG) were higher (or lower) than GC3, GCg and RR of developmental-pivotal genes (DPG) respectively (*** *P* < 0.001, ***P* < 0.01, **P* < 0.05).

*P* values are shown if there was no significance (*P* > 0.05).
