# Supplementary material for: Developmental stage related patterns of codon usage and genomic GC content: searching for evolutionary fingerprints with models of stem cell differentiation
Source: Genome Biol. 2007 Mar 12;8(3):R35. doi: 10.1186/gb-2007-8-3-r35 (PMC1868930; doi:10.1186/gb-2007-8-3-r35)
Supplement: Additional data file 2 — Levels of gene expression are correlated with codon usage, recombination rate, gene length and nucleotide composition [file gb-2007-8-3-r35-S2.doc]

**Additional data file 2:**

**The levels of gene expression are correlated with codon usage, recombination rate, gene length and nucleotide composition**

| **Cell** | **Count** | ***Rs*^1^** | | | | | **Slopes^2^* 1000 (lnEXP)** | | | |
| --- | --- | --- | --- | --- | --- | --- | --- | --- | --- | --- |
|  |  | **RSCUAT3** | **AT3** | **RR** | **CDSLength** | **TransLength** | **T3** | **Ti** | **A3** | **Ai** |
| **ESC** | 2361  A | 0.166  *** | 0.166  *** | -0.033  （0.10） | -0.088  ** | -0.087  ** | 10.60  *** | 5.03  *** | 6.51  *** | 1.19  （0.10） |
| **NSC** | 2395  A | 0.095  *** | 0.098  *** | -0.032  （0.12） | -0.113  *** | -0.099  *** | 6.53  *** | 3.23  ** | 2.57  * | 0.06  （0.93） |
| **HSC** | 2175  A | -0.019  （0.38） | -0.010  （0.65） | -0.011  （0.62） | -0.117  *** | -0.091  ** | 1.38  （0.29） | 0.38  （0.69） | -2.59  （0.06） | -2.47  ** |
| **LVB** | 2577  A | -0.062  ** | -0.056  ** | 0.020  （0.31） | -0.133  *** | -0.100  *** | 2.83^a^  * | 3.42^b^  *** | 4.30^c^  ** | 1.49^d^  * |
| **BM** | 2191  A | -0.043  * | -0.036  （0.09） | -0.010  （0.63） | -0.167  *** | -0.190  *** | 0.91  （0.45） | -1.29  （0.14） | -4.22  ** | -3.24  ** |
| **ESC** | 2963  B | 0.117  *** | 0.112  *** | -0.005  （0.79） | -0.084  *** | -0.097  *** | 7.11  *** | 4.81  *** | 3.03  ** | -0.58  （0.36） |
| **FNSC** | 2770  B | -0.015  （0.42） | -0.014  （0.45） | -0.013  （0.48） | -0.174  *** | -0.157  *** | 1.42  （0.21） | 1.21  （0.14） | -3.93  ** | -2.37  ** |
| **FLHSC** | 2810  B | 0.110  *** | 0.108  *** | -0.021  （0.26） | -0.117  *** | -0.118  *** | 6.78  *** | 4.62  *** | 4.53  ** | 0.04  （0.96） |
| **FLLCP** | 2999  B | 0.123  *** | 0.120  *** | 0.003  （0.85） | -0.172  *** | -0.182  *** | 6.99  *** | 4.77  *** | 4.83  *** | 0.21  （0.76） |
| **FLMBC** | 2834  B | 0.109  *** | 0.109  *** | -0.043  * | -0.147  *** | -0.142  *** | 6.92  *** | 4.32  *** | 3.66  ** | 0.14  （0.84） |

**Additional data file 2 continued**

| **LTHSC** | 1968  B | -0.051  * | -0.041  （0.07） | -0.003  （0.88） | -0.129  *** | -0.110  *** | 0.85  （0.59） | -2.67  * | -5.22  ** | -5.18  *** |
| --- | --- | --- | --- | --- | --- | --- | --- | --- | --- | --- |
| **STHSC** | 1978  B | -0.023  （0.30） | -0.015  （0.50） | 0.011  （0.62） | -0.124  *** | -0.118  *** | 1.66  （0.26） | -1.69  （0.12） | -3.63  * | -4.69  *** |
| **LCP** | 2437  B | 0.086  ** | 0.092  ** | -0.004  （0.84） | -0.163  *** | -0.164  *** | 6.91  *** | 2.41  ** | 2.29  （0.06） | -0.66  （0.36） |
| **MBC** | 2593  B | 0.050  * | 0.056  ** | -0.019  （0.34） | -0.121  *** | -0.084  ** | 5.38  *** | 1.86  * | -0.20  （0.86） | -1.20  （0.10） |
| **CD45** | 2919  B | 0.002  （0.93） | 0.003  （0.87） | 0.015  （0.43） | -0.209  *** | -0.217  *** | 1.18  （0.22） | -0.03  （0.96） | -1.39  （0.16） | -2.89  *** |

1 *Rs:* Spearman correlation coefficient between the levels of gene expression and codon usage (RSCUAT3, AT3), recombination rate (RR), as well as the length of CDS (coding sequence) and transcripts (****P* < 5×10^-6^, ***P* < 0.005, **P* < 0.05). *P* values are shown if there was no significance (*P* > 0.05).

2 Slopes of Ni and N3 with㏑(EXP). Ni and N3 referred to the percentages of nucleotide compositions of synonymous sites in untranslated regions (UTR) and coding sequences (CDS) respectively; a: G3; b: Gi; c: C3; d: Ci; (** *P* < 0.005, **P* < 0.05).  *P* values are shown if there was no significance (*P* > 0.05).
