# Supplementary material for: Developmental stage related patterns of codon usage and genomic GC content: searching for evolutionary fingerprints with models of stem cell differentiation
Source: Genome Biol. 2007 Mar 12;8(3):R35. doi: 10.1186/gb-2007-8-3-r35 (PMC1868930; doi:10.1186/gb-2007-8-3-r35)
Supplement: Additional data file 3 — Fold changes of gene expression are correlated with codon usage, recombination rate and gene length [file gb-2007-8-3-r35-S3.doc]

**Additional data file 3: The fold changes of gene expression are correlated with codon usage, recombination rate and gene length**

| **DP/Model** |  | ***Rs*** | **DP/Model** |  | ***Rs*** | **DP/Model** |  | ***Rs*** |
| --- | --- | --- | --- | --- | --- | --- | --- | --- |
| **ESC/NSC**  **(A)** | RSCUAT3 | 0.176 *** | **ESC/FNSC**  **(B)** | RSCUAT3 | 0.249 *** | **FLHSC/**  **LTHSC**  **(B)** | RSCUAT3 | 0.143 *** |
|  | AT3 | 0.175 *** |  | AT3 | 0.238 *** |  | AT3 | 0.136 *** |
|  | RR | 0.006 （0.81） |  | RR | -0.014 (0.49) |  | RR | -0.002 (0.93) |
|  | CDS | 0.066 ** |  | CDS | 0.146 *** |  | CDS | -0.048 * |
|  | Trans | 0.017 （0.47） |  | Trans | 0.074 ** |  | Trans | -0.142 *** |
| **NSC/LVB**  **(A)** | RSCUAT3 | 0.387 *** | **ESC/FLHSC**  **(B)** | RSCUAT3 | 0.012 (0.56) | **LTHSC/**  **STHSC**  **(B)** | RSCUAT3 | -0.089 ** |
|  | AT3 | 0.378 *** |  | AT3 | 0.003 (0.90) |  | AT3 | -0.086 ** |
|  | RR | -0.083 ** |  | RR | 0.003 (0.86) |  | RR | -0.005 (0.85) |
|  | CDS | 0.051 * |  | CDS | 0.038 (0.06) |  | CDS | 0.085 ** |
|  | Trans | 0.011 （0.62） |  | Trans | 0.024 (0.24) |  | Trans | 0.179 *** |
| **ESC/HSC**  **(A)** | RSCUAT3 | 0.341 *** | **FLHSC/**  **FLLCP**  **(B)** | RSCUAT3 | 0.046 * | **STHSC/**  **LCP**  **(B)** | RSCUAT3 | -0.144 *** |
|  | AT3 | 0.338 *** |  | AT3 | 0.058 ** |  | AT3 | -0.141 *** |
|  | RR | -0.045 (0.07) |  | RR | -0.037 (0.06) |  | RR | 0.034 (0.14) |
|  | CDS | 0.034 (0.17) |  | CDS | 0.158 *** |  | CDS | 0.150 *** |
|  | Trans | 0.005 (0.85) |  | Trans | 0.205 *** |  | Trans | 0.170 *** |
| **HSC/BM**  **(A)** | RSCUAT3 | -0.043 (0.08) | **FLLCP/**  **FLMBC**  **(B)** | RSCUAT3 | -0.103 *** | **LCP/MBC**  **(B)** | RSCUAT3 | 0.081 ** |
|  | AT3 | -0.043 (0.08) |  | AT3 | -0.108 *** |  | AT3 | 0.081 ** |
|  | RR | 0.018 (0.46) |  | RR | 0.062 ** |  | RR | 0.008 (0.71) |
|  | CDS | 0.086 ** |  | CDS | -0.041 * |  | CDS | -0.087 ** |
|  | Trans | 0.105 ** |  | Trans | -0.059 ** |  | Trans | -0.197 *** |

*Rs:* Spearman correlation coefficient between the fold change of gene expression (FC) and codon usage (RSCUAT3, AT3), recombination rate (RR), as well as the length of CDS (coding sequence) and transcripts (Trans) (****P* < 5×10^-6^, ***P* < 0.005, **P* < 0.05). *P* values are shown if there was no significance (*P* > 0.05).
