# Supplementary material for: Developmental stage related patterns of codon usage and genomic GC content: searching for evolutionary fingerprints with models of stem cell differentiation
Source: Genome Biol. 2007 Mar 12;8(3):R35. doi: 10.1186/gb-2007-8-3-r35 (PMC1868930; doi:10.1186/gb-2007-8-3-r35)
Supplement: Additional data file 4 — GC3 and GCg of developmental-pivotal genes, developmental-specific genes and both together in each differentiation pair [file gb-2007-8-3-r35-S4.doc]

**Additional data file 4: the GC3 and GCg of DPG, DSG and DPG+DSG in each differentiation pair**

| **DP** | **DPG** | **Count** | **GC3** | **GCg** | **DSG** | **Count** | **GC3** | **GCg** | **DPG+DSG** | **GC3** | **GCg** |
| --- | --- | --- | --- | --- | --- | --- | --- | --- | --- | --- | --- |
| **ESC/NSC** | FC>2 | 178 | 0.522  *** | 0.437  *** | ESC | 120 | 0.607  * | 0.448  *** | 298 | 0.556  *** | 0.441  *** |
|  | FC<0.5 | 296 | 0.584 | 0.454 | NSC | 187 | 0.642 | 0.469 | 483 | 0.607 | 0.460 |
| **NSC/LVB** | FC>2 | 578 | 0.510  *** | 0.433  *** | NSC | 89 | 0.580  *** | 0.453  (0.10) | 667 | 0.520  *** | 0.436  *** |
|  | FC<0.5 | 122 | 0.636 | 0.461 | LVB | 142 | 0.635 | 0.462 | 264 | 0.635 | 0.462 |
| **ESC/HSC** | FC>2 | 432 | 0.505  *** | 0.431  *** | ESC | 187 | 0.596  *** | 0.447  *** | 619 | 0.533  *** | 0.435  *** |
|  | FC<0.5 | 224 | 0.610 | 0.469 | HSC | 226 | 0.646 | 0.472 | 450 | 0.628 | 0.470 |
| **HSC/BM** | FC>2 | 236 | 0.592  * | 0.459  (0.08) | HSC | 141 | 0.638  ** | 0.473  ** | 377 | 0.609  ** | 0.464  *** |
|  | FC<0.5 | 145 | 0.565 | 0.451 | BM | 85 | 0.593 | 0.454 | 230 | 0.576 | 0.452 |
| **ESC/FNSC** | FC>2 | 459 | 0.528  *** | 0.442  *** | ESC | 498 | 0.599  *** | 0.455  (0.13) | 957 | 0.565  *** | 0.449  *** |
|  | FC<0.5 | 429 | 0.598 | 0.454 | FNSC | 299 | 0.635 | 0.460 | 728 | 0.614 | 0.456 |
| **ESC/FLHSC** | FC>2 | 445 | 0.566  (0.50) | 0.450  (0.09) | ESC | 519 | 0.608  (0.32) | 0.456  (0.44) | 964 | 0.589  （0.31） | 0.454  （0.06） |
|  | FC<0.5 | 367 | 0.571 | 0.447 | FLHSC | 363 | 0.617 | 0.455 | 730 | 0.593 | 0.451 |
| **FLHSC/FLLCP** | FC>2 | 129 | 0.572  (0.17) | 0.446  * | FLHSC | 151 | 0.600  * | 0.447  ** | 280 | 0.587  ** | 0.446  *** |
|  | FC<0.5 | 150 | 0.587 | 0.458 | FLLCP | 343 | 0.624 | 0.460 | 493 | 0.613 | 0.459 |

**Additional data file 4 continued:**

| **FLLCP/FLMBC** | FC>2 | 364 | 0.602  ** | 0.459  ** | FLLCP | 434 | 0.631  *** | 0.465  *** | 798 | 0.618  *** | 0.462  *** |
| --- | --- | --- | --- | --- | --- | --- | --- | --- | --- | --- | --- |
|  | FC<0.5 | 243 | 0.575 | 0.449 | FLMBC | 265 | 0.594 | 0.448 | 508 | 0.585 | 0.449 |
| **FLHSC/LTHSC** | FC>2 | 304 | 0.537  *** | 0.445  (0.82) | FLHSC | 935 | 0.606  (0.77) | 0.463  *** | 1239 | 0.589  （0.58） | 0.458  *** |
|  | FC<0.5 | 228 | 0.587 | 0.445 | LTHSC | 93 | 0.607 | 0.439 | 321 | 0.593 | 0.444 |
| **LTHSC/STHSC** | FC>2 | 42 | 0.567  (0.24) | 0.437  (0.22) | LTHSC | 182 | 0.590  (0.92) | 0.446  ** | 224 | 0.586  （0.31） | 0.445  ** |
|  | FC<0.5 | 86 | 0.536 | 0.448 | STHSC | 192 | 0.585 | 0.459 | 278 | 0.569 | 0.456 |
| **STHSC/LCP** | FC>2 | 91 | 0.599  *** | 0.449  (0.16) | STHSC | 79 | 0.599  (0.99) | 0.455  (0.44) | 170 | 0.599  （0.28） | 0.452  （0.38） |
|  | FC<0.5 | 130 | 0.544 | 0.441 | LCP | 538 | 0.600 | 0.459 | 668 | 0.589 | 0.455 |
| **LCP/MBC** | FC>2 | 353 | 0.542  *** | 0.443  * | LCP | 225 | 0.606  (0.68) | 0.456  (0.64) | 578 | 0.567  *** | 0.448  ** |
|  | FC<0.5 | 252 | 0.584 | 0.450 | MBC | 384 | 0.610 | 0.457 | 636 | 0.599 | 0.454 |

Wilcoxon test was used to determine whether GC3 and GCg were different in a particular differentiation pair between developmental-pivotal genes (DPGs) enriched in earlier (FC>2) and later (FC < 0.5) stages, as well as between developmental-specific genes (DSGs) enriched in the earlier and later stages or between pooled DPGs and DSGs in the earlier and later stages (for example, FNSC referred to developmental specific genes in FNSC of differentiation pair ESC/FNSC (****P* < 0.001, ***P* < 0.01, **P* < 0.05). *P* values are still shown if there was no significance (*P* > 0.05).
