# Supplementary material for: Developmental stage related patterns of codon usage and genomic GC content: searching for evolutionary fingerprints with models of stem cell differentiation
Source: Genome Biol. 2007 Mar 12;8(3):R35. doi: 10.1186/gb-2007-8-3-r35 (PMC1868930; doi:10.1186/gb-2007-8-3-r35)
Supplement: Additional data file 5 — Comparisons of substitution rates between developmental-pivotal genes and developmental-specific genes [file gb-2007-8-3-r35-S5.doc]

**Additional data file 5: The Comparisons of substitution rates between DPG and DSG**

| **DP^1^/Model** | **Class** | | **Ka** | **Ks** | **Ka/Ks** | **Ks_noDS** |
| --- | --- | --- | --- | --- | --- | --- |
| **ESC/NSC**  **(A)** | **ESC** | **DPG** | 0.049  *** | 0.555  *** | 0.084  *** | 0.584  *** |
|  |  | **DSG** | 0.097 | 0.627 | 0.136 | 0.703 |
|  | **NSC** | **DPG** | 0.044  ** | 0.545  (0.11) | 0.075  ** | 0.580  (0.07) |
|  |  | **DSG** | 0.058 | 0.563 | 0.101 | 0.619 |
| **NSC/LVB**  **(A)** | **NSC** | **DPG** | 0.042  * | 0.548  * | 0.074  (0.08) | 0.573  * |
|  |  | **DSG** | 0.050 | 0.583 | 0.068 | 0.632 |
|  | **LVB** | **DPG** | 0.042  *** | 0.549  ** | 0.069  *** | 0.589  * |
|  |  | **DSG** | 0.081 | 0.592 | 0.123 | 0.652 |
| **ESC/HSC**  **(A)** | **ESC** | **DPG** | 0.042  *** | 0.542  *** | 0.072  *** | 0.565  *** |
|  |  | **DSG** | 0.074 | 0.603 | 0.107 | 0.663 |
|  | **HSC** | **DPG** | 0.052  *** | 0.547  ** | 0.082 *** | 0.583  ** |
|  |  | **DSG** | 0.086 | 0.593 | 0.133 | 0.647 |
| **HSC/BM**  **(A)** | **HSC** | **DPG** | 0.065  (0.45) | 0.590  (0.11) | 0.099  (0.45) | 0.627  (0.400 |
|  |  | **DSG** | 0.070 | 0.567 | 0.104 | 0.639 |
|  | **BM** | **DPG** | 0.067  *** | 0.576  (0.07) | 0.107  *** | 0.609  * |
|  |  | **DSG** | 0.096 | 0.615 | 0.148 | 0.670 |
| **ESC/FNSC**  **(B)** | **ESC** | **DPG** | 0.045  *** | 0.560  ** | 0.081  *** | 0.590  *** |
|  |  | **DSG** | 0.083 | 0.591 | 0.125 | 0.642 |
|  | **FNSC** | **DPG** | 0.053  * | 0.550  * | 0.088  * | 0.580  ** |
|  |  | **DSG** | 0.062 | 0.573 | 0.100 | 0.630 |
| **ESC/FLHSC**  **(B)** | **ESC** | **DPG** | 0.046  *** | 0.576  (0.33) | 0.073  *** | 0.606  (0.09) |
|  |  | **DSG** | 0.072 | 0.571 | 0.113 | 0.625 |
|  | **FLHSC** | **DPG** | 0.060  *** | 0.570  * | 0.099  *** | 0.600  * |
|  |  | **DSG** | 0.097 | 0.599 | 0.143 | 0.640 |

**Additional data file 5 continued:**

| **FLHSC/FLLCP**  **(B)** | **FLHSC** | **DPG** | 0.068  (0.06) | 0.563  (0.43) | 0.107  (0.08) | 0.607  (0.23) |
| --- | --- | --- | --- | --- | --- | --- |
|  |  | **DSG** | 0.082 | 0.564 | 0.123 | 0.590 |
|  | **FLLCP** | **DPG** | 0.069  (0.19) | 0.594  (0.11) | 0.104  (0.07) | 0.629  (0.34) |
|  |  | **DSG** | 0.068 | 0.570 | 0.110 | 0.614 |
| **FLLCP/FLMBC**  **(B)** | **FLLCP** | **DPG** | 0.062  ** | 0.602  (0.05) | 0.096  ** | 0.631  (0.49) |
|  |  | **DSG** | 0.075 | 0.581 | 0.116 | 0.634 |
|  | **FLMBC** | **DPG** | 0.068  *** | 0.566  ** | 0.107  ** | 0.616  * |
|  |  | **DSG** | 0.088 | 0.600 | 0.135 | 0.652 |
| **FLHSC/LTHSC**  **(B)** | **FLHSC** | **DPG** | 0.055  ** | 0.578  (0.43) | 0.084  *** | 0.583  ** |
|  |  | **DSG** | 0.066 | 0.579 | 0.103 | 0.622 |
|  | **LTHSC** | **DPG** | 0.075  （0.20） | 0.567  (0.24) | 0.108  (0.16) | 0.603  (0.29) |
|  |  | **DSG** | 0.074 | 0.586 | 0.112 | 0.620 |
| **LTHSC/STHSC**  **(B)** | **LTHSC** | **DPG** | 0.084  (0.23) | 0.563  (0.33) | 0.128  (0.35) | 0.632  (0.13) |
|  |  | **DSG** | 0.063 | 0.564 | 0.106 | 0.595 |
|  | **STHSC** | **DPG** | 0.071  (0.31) | 0.614  (0.06) | 0.107  (0.45) | 0.634  (0.29) |
|  |  | **DSG** | 0.066 | 0.575 | 0.109 | 0.618 |
| **STHSC/LCP**  **(B)** | **STHSC** | **DPG** | 0.076  (0.50) | 0.562  * | 0.123  (0.29) | 0.615  (0.37) |
|  |  | **DSG** | 0.083 | 0.610 | 0.104 | 0.605 |
|  | **LCP** | **DPG** | 0.070  (0.45) | 0.590  (0.29) | 0.106  (0.37) | 0.606  (0.27) |
|  |  | **DSG** | 0.063 | 0.578 | 0.103 | 0.617 |
| **LCP/MBC**  **(B)** | **LCP** | **DPG** | 0.054  *** | 0.580  (0.22) | 0.086  *** | 0.596  * |
|  |  | **DSG** | 0.072 | 0.589 | 0.120 | 0.626 |
|  | **MBC** | **DPG** | 0.066  * | 0.570  (0.09) | 0.103  * | 0.610  (0.11) |
|  |  | **DSG** | 0.081 | 0.590 | 0.124 | 0.634 |

1 DP: differentiation pairs

2 Wilcoxon test was used to determine whether Ka, Ks, Ka/Ks and Ks_noDS of developmental-specific genes (DSG) were higher (or lower) than Ka, Ks, Ka/Ks and Ks_noDS of developmental-pivotal genes (DPG) respectively (*** *P* < 0.001, ***P* < 0.01, **P* < 0.05).

*P* values are shown if there was no significance (*P* > 0.05).
